# On the Benefits of Listen before Talk Scheme for NB-Fi Networks

**DOI:** 10.3390/s23229054

**Published:** 2023-11-08

**Authors:** Irina Pavlova, Dmitry Bankov, Evgeny Khorov, Andrey Lyakhov

**Affiliations:** Institute for Information Transmission Problems of the Russian Academy of Sciences, 127051 Moscow, Russia; pavlova@wireless.iitp.ru (I.P.); khorov@iitp.ru (E.K.); lyakhov@iitp.ru (A.L.)

**Keywords:** NB-Fi, ultra-narrow band, Carrier Sense Multiple Access, Listen Before Talk, performance evaluation, energy consumption

## Abstract

NB-Fi (Narrow Band Fidelity) is a promising protocol for low-power wide-area networks. NB-Fi networks use license-exempt Industrial, Scientific, and Medical (ISM) bands and, thus, NB-Fi devices can work in two modes: with and without Listen Before Talk (LBT). This paper compares these modes with different implementations of LBT in terms of packet loss rate (PLR), delay, energy consumption, and throughput. Interestingly, in some scenarios, the results contradict expectations from the classic papers on channel access because of the peculiarities of the NB-Fi technology. These contradictions are explained in the paper. The results show that LBT can significantly improve all the considered performance indicators when the network load exceeds 40 packets per second. With extensive simulation, we show that in a small NB-Fi network, the optimal PLR, delay, and energy consumption are obtained with the implementation of LBT that corresponds to non-persistent CSMA. In a large NB-Fi network, where some devices can be hidden from others, the best strategy to improve PLR, delay, throughput, and energy consumption is to use the implementation of LBT that corresponds to p-persistent CSMA.

## 1. Introduction

Low-power wide-area networks (LPWANs) [1] are an important part of the Internet of Things, which, according to forecasts, will serve approximately 14% of all Internet connections [2] by 2023. NB-Fi (Narrow Band Fidelity) [3,4] is a rather new and not so well studied LPWAN protocol widely deployed in several countries for environmental monitoring, data collection for smart homes and utilities, urban planning, and infrastructure management. NB-Fi networks operate in France [5], Serbia [6], Argentina [7], Moldova [8], Kazakhstan [9], and Russia [10]. Thus, the study of the benefits and the limits of this protocol is necessary to use this technology effectively and to satisfy the quality of service requirements in different scenarios.

NB-Fi networks use license-exempt Industrial, Scientific, and Medical (ISM) bands; thus, for channel access, NB-Fi devices must abide by several restrictions imposed by the regulator. By default, NB-Fi devices do not sense the channel before transmission, i.e., they use the Aloha channel access method [11]. This channel access method can be easily implemented and is suitable for cheap Internet of Things (IoT) devices, but it forces devices to limit their duty cycle, i.e., the share of used channel time. Another possible channel access method is Listen Before Talk (LBT), also known as Carrier Sense Multiple Access (CSMA), when devices sense the channel before packet transmission. Implementation of the LBT mode is more complex, but, according to the classic research on CSMA [12], it provides a better packet delivery rate, and different implementations of LBT are often used in wireless networks [13,14]. However, LBT introduces additional energy consumption, which is crucial for battery-supplied IoT devices. Thus, the motivation of our work is to investigate whether the properly configured LBT mode in NB-Fi networks can potentially improve the network performance and the quality of service of traffic in IoT scenarios.

The LBT implementation described in the NB-Fi standard [3] corresponds to persistent CSMA, where the device listens to the channel and, if the channel is busy, should postpone transmission until it becomes idle. However, from the classic studies of CSMA, we know that persistent CSMA is not very efficient for wireless networks [12]. At the same time, although LBT or CSMA have been studied in theoretical works and recent technology-oriented papers, the existing results are not completely relevant to NB-Fi because these studies do not consider the peculiarities of this technology. Despite its importance, no studies have considered the LBT mode in NB-Fi, except for our previous work [15], where we compare the energy consumption of NB-Fi devices for LBT and non-LBT modes. Although energy consumption is an important indicator for IoT devices, which are often powered by batteries or low-power renewable sources of energy such as solar panels [16], the study [15] of LBT in NB-Fi networks does not consider other performance indicators such as PLR, delay and throughput, which are also important for IoT scenarios [17,18]. Thus, the goal of our work is to close this gap and determine the most suitable implementation of LBT for NB-Fi networks to improve the PLR, delay, throughput, and energy consumption compared with the default non-LBT operation mode and with the straightforward implementation of LBT as it is described in the standard.

In this paper, we extend the study of LBT in NB-Fi by accurately taking into account all the features of NB-Fi and comparing such important metrics as the PLR, delay, throughput, and energy consumption of NB-Fi devices when using channel access without LBT and when using different types of LBT. Moreover, we refine the sensors’ energy consumption in different states based on the information provided by the vendor and thus obtain more accurate results than in [15].

The contribution of this work is that we evaluate the performance of NB-Fi networks with different implementations of LBT and determine those that provide the best PLR, delay, throughput, and energy consumption. We consider two scenarios: a scenario with a small network, where most devices can sense transmissions of each other, and a scenario with a large network, where devices can be hidden from each other. Based on our studies, we develop recommendations on which type of LBT to use in each scenario.

The rest of the paper is organized as follows. In Section 2, we review existing solutions related to NB-Fi and the LBT mode. In Section 3, we describe the most relevant features of NB-Fi. Section 4 introduces the CSMA algorithms used in the paper. Section 5 describes the considered scenario, and Section 6 states the problem. Section 7 presents and discusses the numerical results. Section 8 concludes the paper.

## 2. Related Works

At the moment, most papers about NB-Fi found in the literature present reviews of the technology [19,20], or consider only the non-LBT operation mode [4,21]. One study [22] evaluates the packet loss rate, packet error rate, and average delay in the non-LBT mode of NB-Fi, Sigfox, and LoRaWAN networks in different scenarios and shows the efficiency of NB-Fi for the reliable delivery of small packets.

At the same time, to the best of our knowledge, only our initial work [15] studies the energy consumption of NB-Fi devices, with different LBT schemes. In our previous paper, we did not consider other performance indicators relevant to LPWANs such as packet loss rate (PLR), network throughput, and delay, and the goal of this paper is to cover this gap. Moreover, in our new paper, we refine the sensors’ energy consumption in different states based on the information provided by the vendor.

Many studies, including the classic ones, consider LBT and non-LBT modes for networks of different technologies [12,23,24,25,26,27], but they do not include any comparison of the device’s energy consumption and do not consider the peculiarities of NB-Fi related to channel access and the possibility of data transmission at different rates.

Many papers [28,29,30,31,32,33] are dedicated to the study of LBT in LoRa/LoRaWAN networks, which belong to the same—LPWAN—class of networks as NB-Fi. Papers [28,29] compare the device energy consumption in the Aloha and “non-persistent CSMA” modes for LoRaWAN networks. Paper [28] shows that the LBT mode achieves a higher packet delivery probability than the Aloha mode, but it is less energy efficient. According to [29], this result holds only for an underutilized network, while in a network consisting of many devices, LBT reduces the devices’ energy consumption. However, [29] neither studies other types of CSMA nor determines the most efficient implementation of LBT for LPWANs. In [30], the authors propose to use *p*-persistent CSMA in LoRaWAN and evaluate the Packet Reception Ratio. It is shown that the smallest value of the persistence parameter provides the best results in all scenarios. However, the authors consider scenarios with only a few devices (e.g., 20, 40, 60, 80), and it is unclear how their solution performs in typical IoT scenarios with a large numbers of devices.

Papers [31,32,33] develop new LBT mechanisms for LoRa. Although the results demonstrate that the proposed protocols significantly improve channel efficiency, the considered algorithms differ from the classic types of CSMA, which we consider in the paper. Moreover, [31,33] do not compare CSMA modes with Aloha.

To sum up, many papers present in the literature study the LBT mode for LPWAN technologies, but they do not consider NB-Fi, see Table 1. Most of the LBT research among LPWAN technologies is devoted to LoRaWAN, which has many differences from NB-Fi on the physical layer [22], and that is why their results are not suitable for NB-Fi. In addition, the existing studies do not consider the performance of the LBT mode in networks consisting of thousands of devices, while in IoT networks, the number of devices is expected to be even larger. Thus, the novelty of this paper is that it is the first to evaluate PLR, delay, and throughput, taking into account the peculiarities of NB-Fi networks for different LBT schemes, and then to develop recommendations as to which LBT scheme to use depending on the scenario of NB-Fi use.

## 3. A Short Description of NB-Fi

In this section, we describe the most relevant details of NB-Fi, while the detailed description of NB-Fi can be found in [4,15].

An NB-Fi network has a “star” topology and consists of a server, base stations (BSs), and end devices (hereinafter called sensors). Typically, sensors transmit their data frames via the wireless channel to a BS, which forwards the frames to the server through a wired link. In the reverse direction, the server sends service frames, e.g., acknowledgments or data.

In many countries, NB-Fi networks usually use the 868.7–869.2 MHz ISM band and have separate uplink and downlink channels. The uplink channel is at least 51.2 kHz wide, while the downlink channel is at least 102.4 kHz wide.

NB-Fi devices usually use differential binary phase-shift keying (DBPSK) with data rates of 50, 400, 3200, and 25,600 bps. All frames in NB-Fi have the same size of 288 bits, so their duration and the width of the subchannel required for transmission are determined by the used data rate (see Table 2). Note that in NB-Fi, all data rates have the same spectral efficiency (bits/Hz/s): the lower ones have higher reliability because of narrower bands and, consequently, higher signal power spectral density.

By default, sensors access the channel according to an Aloha-like scheme: a sensor does not sense the channel before transmission and sends the frame immediately if it is the first transmission attempt, or after a random delay before a retry. Sensors can also use the LBT mode: before transmitting a packet in some frequency band, the sensor evaluates the signal strength in this band to ensure that no other devices are transmitting. If the sensor does not detect any transmission, it transmits its packet, otherwise, the sensor postpones its transmission until the signal strength falls below a threshold.

To transmit a frame, each sensor selects a subchannel within the uplink channel. Effectively, the central frequency of the subchannel is randomized within the allocated frequency band for each frame transmission attempt, including the retries. This rule has an exception if the required subchannel width is comparable to the channel width (e.g., the data rate is 25,600 bps, while the channel width is 51.2 kHz). In this case, the central frequency of the subchannel equals the central frequency of the channel.

The subchannel for downlink transmissions is determined by the sensor’s identifier and does not change at different transmission attempts.

In this paper, we consider that sensors operate in discontinuous reception (DRX) mode, and their frames require acknowledgment. In this mode, after a frame transmission, the sensor waits for $T_{delay}$ and listens to the downlink channel until it either receives an acknowledgment or a $T_{listen}$ interval passes (see Table 2). If no acknowledgment is received, the sensor retries after a random time distributed uniformly over the interval $\left(0,T_{rnd}\right)$. Retransmissions are performed until the reception of an acknowledgment or upon reaching the configurable retry limit.

## 4. LBT Variants

**Figure 1:**
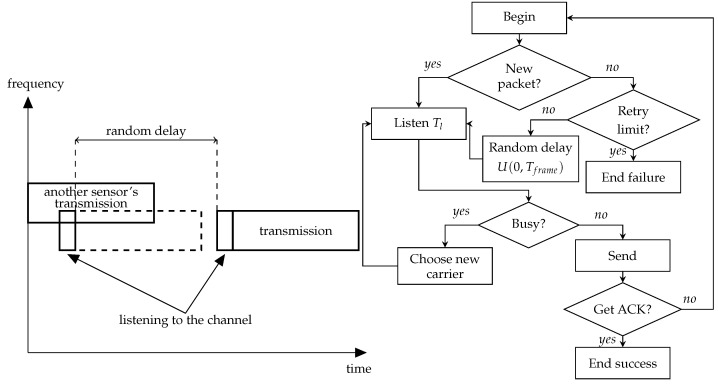
Schemes of the non-persistent CSMA.

**Figure 2:**
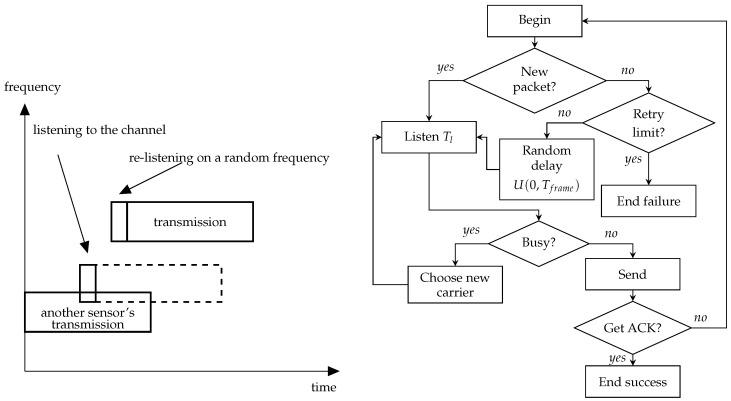
Schemes of the non-persistent CSMA with frequency hopping.

**Figure 3:**
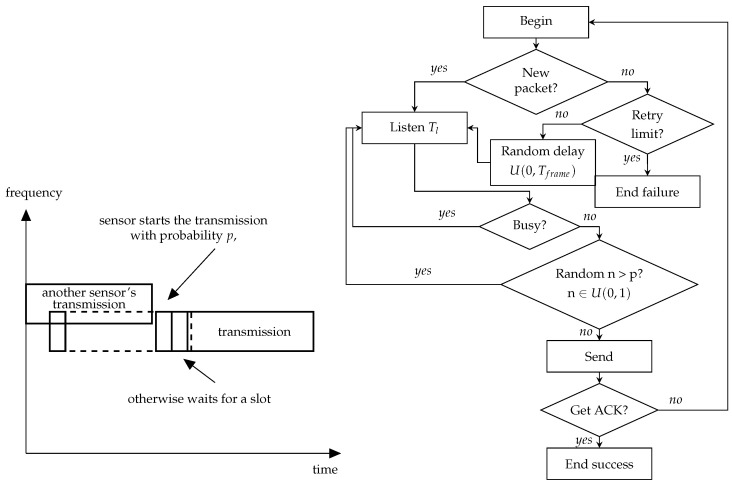
Schemes of the *p*-persistent CSMA.

The NB-Fi specification mentions that devices can operate in LBT mode but does not provide much detail on how it should work. The straightforward interpretation of the description of LBT in the standards means that the device listens to the channel before transmission and, if the power of the signal in the channel is greater than some level, it should wait until the channel becomes idle, and only then should it start the transmission. Such operation of LBT corresponds to the persistent CSMA algorithm. Apart from this algorithm, we consider other modes of sensors’ operation with LBT that implement different CSMA algorithms, see Figure 1, Figure 2 and Figure 3. Namely, we consider:

- Non-persistent CSMA;
- Non-persistent CSMA with frequency hopping;
- *p*-persistent CSMA.

They all have in common that, before transmission, the sensor listens to the channel and compares the power of the signal with a threshold. If the signal is greater than the threshold, the channel is considered busy, and the sensor performs different actions depending on the used algorithm. Let us describe the considered types of CSMA in more detail.

In the non-persistent CSMA mode, the sensor selects a random subchannel when a packet arrives, see Figure 1, and senses the subchannel for the σ time interval. The specification does not define the value of σ, but it is reasonable to make it equal to the symbol duration at the used data rate (see Table 3) because of two reasons. First, this value is sufficient to estimate the signal power. Second, this value is enough for the signal to propagate between any sensors within their transmission range. If the signal strength is below the sensitivity threshold *S* (see Table 3), the channel is considered idle, and the sensor transmits the packet. Otherwise, the channel is considered busy, and the sensor repeatedly senses the channel with a random delay between the sensing attempts until the channel becomes idle. The distribution of this delay is not specified in the standard, so we choose it uniformly from the interval $\left[0,T_{Frame}\right]$, where $T_{Frame}$ is the frame duration at the used data rate. Although the interval for the random delay seems quite narrow, we choose such an interval because preliminary results show that the further increase in its upper bound does not provide any significant improvement in the PLR or energy consumption.

A variation of the non-persistent CSMA mode is implemented in the non-persistent CSMA mode with frequency hopping, see Figure 2. In this mode, when the sensor finds the selected subchannel busy, it senses another random subchannel, chosen equiprobably from the available frequency band.

In the *p*-persistent CSMA mode, when the sensor generates a data frame, it senses the subchannel for a σ time interval, see Figure 3. In the case of an idle subchannel, the sensor transmits the frame. Otherwise, the sensor waits until the subchannel becomes idle. Then, it transmits the packet with the probability *p* and repeats listening to the subchannel within the time interval σ with the probability 1 − *p*. We further denote a special case of *p*-persistent CSMA with parameter p=1 as persistent CSMA.

## 5. Studied Scenario

We consider a network that consists of a server, 1000 sensors evenly distributed in a circle of radius *R*, and a BS located in the center of this circle. The widths of the uplink and downlink channels are 51.2 kHz and 102.4 kHz, respectively. The sensors generate a Poisson flow of frames with a load λ and transmit them to BS. To transmit their frames, sensors use the assigned data rate, and the BS uses the same data rate for acknowledgments. A frame is discarded after seven unsuccessful transmission attempts. Sensors have a buffer that can store up to one frame, and new frames preempt the old ones when they are generated and the buffer is occupied.

We use the Okumura–Hata model [35] to describe the signal propagation in the uplink channel, and the model from [36] to describe the propagation between sensors. We choose such models because we assume that the sensors are located at the same height, while the BS is at an elevation of 30 m higher than the sensors. Thus, to calculate the signal power at the receiver, we consider that the sensor or the base station transmits the frame with the power of $P_{o}=14\;\mathrm{dBm}$, and, at the receiver, the power of this signal equals $P_{o}-PL(d)$, where *d* is the distance between the transmitter and the receiver, and PL(d) is the path loss corresponding to such a distance. We use function PL(d) from [35] if the transmitter or the receiver is BS, and from [36] if both the transmitter and the receiver are sensors. The BS receives a frame if the signal to interference and noise ratio (SINR) during its transmission is not less than 7 dB (such an SINR corresponds to a bit error rate of $10^{-5}$ [4]). Otherwise, the frame is damaged. Table 3 provides the maximal values for the receiving sensitivity *S*, BS reception distance $R_{base}$, and the distance $R_{sensor}$ at which sensors can sense each others’ signals.

The sensors’ energy consumption depends on their state, see Table 4. We consider that the sensor can be in idle, receiving, or transmitting states. In the idle state, the sensor’s transceiver is turned off and does not consume additional power. In the receiving state, the sensor can listen to the channel, determine if the channel is idle, and receive packets. In the receiving state, the sensor consumes $P_{rx}$ power. In the transmitting state, the sensor transmits its frame and consumes $P_{tx}$ power. According to [34], a sensor is powered by a voltage *V* and consumes a current $I_{rx}$ in the receiving state and a current $I_{tx}$ in the transmitting one. In its turn, the power source consumes $I_{s}$ current. Thus, we obtain that the sensor consumes $P_{tx}=\left(I_{tx}+I_{s}\right)\times V$ power when transmitting data and $P_{rx}=\left(I_{rx}+I_{s}\right)\times V$ when receiving or listening to the channel in LBT mode and when waiting for an acknowledgment. Note that the resulting power consumption values differ from those used in [15] and describe the devices more correctly. Here we consider only the power consumed by the sensor’s transceiver and leave the other sources of power consumption out of consideration because they are the same for all the considered kinds of LBT and non-LBT modes.

## 6. Problem Statement

Although the performances of Aloha and different CSMA modes have been compared in classic works [12], it is not obvious which mode is better for NB-Fi in typical IoT scenarios such as the one described in this section. First, the classic results do not consider the possibility of NB-Fi using different data rates, the transmissions of which require different subchannel widths and thus have different probabilities to resolve conflicts in the frequency domain. Second, the classic results do not consider the small buffers at the end devices, which are typical for low-memory sensors. Small buffers significantly increase the loss probability at high loads. At the same time, packets lost due to buffer overflow do not contribute to the energy consumption and the average delay. For the described scenario, we state the problem to compare different operation modes: Aloha and the listed CSMA types in terms of device energy consumption, PLR, latency, and throughput, and to provide recommendations on which operation mode to use considering the peculiarities of NB-Fi networks.

## 7. Numerical Results

We simulate the scenario described in Section 6 to evaluate the dependence of PLR, the average delay per successfully transmitted packet, throughput, and the normalized sensor’s energy consumption *E* per a successfully transmitted packet on the load. In the simulation, we assume that sensors operate independently, have equal capabilities and parameters, such as the transmission power of 14 dBm, and use the same data rate assigned by the BS. We assume that a transmitted frame can be damaged only by a collision with other frames. Thus, a frame is received only if the SINR during the frame transmission exceeds 7 dB for the whole duration of the frame, otherwise, it is lost. We assume that the sensors are static, and the channel is static and flat as well, i.e., we neglect the frequency selectivity.

We measure throughput as the ratio of the number of successful packets sent by the sensors during the experiment to the experiment time. Furthermore, we calculate *E* as the total energy consumed by the sensors during the simulation according to the model described in Section 6 divided by the number of successfully delivered frames. We calculate the delay of frame transmission as the time interval between the frame generation at the sensor and the successful delivery of the frame to the BS. Please note that the delay is calculated only for the successful frames. The average delay is obtained by averaging this value over all sensors in the network and all their delivered frames. For each value of λ, we perform 100 runs of the experiment.

### 7.1. Results for a Circle with Radius of 0.4 km

Let us first study the network performance in the case of R= 0.4 km radius. In such a network, all sensors can sense each other’s transmission (see Table 3).

#### 7.1.1. PLR

**Figure 4:**
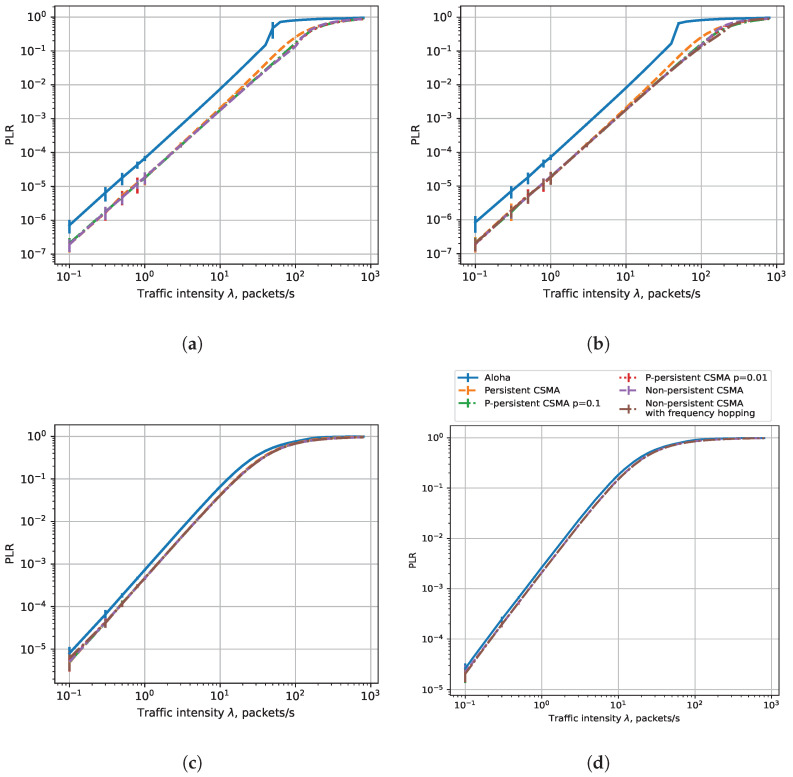
PLR, R = 0.4 km, rates: (**a**) 25,600 bps, (**b**) 3200 bps, (**c**) 400 bps, (**d**) 50 bps.

Figure 4 shows the dependencies of PLR on the load for different data rates and six sensor operating modes: Aloha, non-persistent CSMA, persistent CSMA, non-persistent CSMA with frequency hopping, and *p*-persistent CSMA with p= 0.1 and p= 0.01. Interestingly, depending on the data rate, the result may align with or contradict the conclusions of the classic works on CSMA because of the peculiarities of the NB-Fi technology. Let us consider them in detail.

Figure 4a,b show results for the 3.2 kbps and 25.6 kbps rates. We do not show the dependence for non-persistent CSMA with frequency hopping for the data rate of 25.6 kbps: with such a bitrate and the 51.2 kHz-wide uplink channel, all sensors transmit in the same subchannel located in the center of the channel. For the 3.2 kbps and 25.6 kbps data rates, the PLR values for CSMA modes are significantly lower than for the Aloha mode, which aligns with the classic results [12]. At these data rates, CSMA efficiently avoids collisions and thus decreases the PLR. Another result predicted from classic CSMA analysis is that at low loads, the difference in the PLR between various CSMA modes is hardly noticeable because, to avoid collision, it is enough not to transmit while the channel is occupied, which is what sensors do in all the considered CSMA modes. However, at high loads, we see a significant difference in the performance of various types of CSMA. The persistent CSMA is the most inefficient because in this mode, a sensor listens to the channel and waits until it becomes idle, but when several other sensors generate frames during transmission and plan to transmit in overlapping subchannels, they start their transmissions simultaneously after the end of the current transmission, and a collision occurs. The other CSMA modes do not have such a drawback and thus achieve a slightly lower PLR. Let us also note that at a load greater than 100 packets per second, the PLR comes close to 1, which is caused by the high contention for channel access and the limitations of the buffers at the sensors. Specifically, if the sensor cannot transmit its frame as soon as it is generated, at a high load, the frame with a high probability is preempted by a newly generated frame and thus is lost.

Figure 4c,d show the results for the data rates of 50 and 400 bps. We can see that the PLR of different types of CSMA barely differs from the PLR in Aloha mode. Such a result contradicts the classic results of studies of CSMA and Aloha but is explained by the peculiarities of the NB-Fi technology. Specifically, at such data rates, the transmissions use very narrow subchannels, and the sensors rarely select overlapping subchannels. Therefore, listening to the channel only provides a tiny gain in PLR over the Aloha mode.

From the obtained results, we can conclude that the lowest PLR can be obtained with p-persistent or non-persistent CSMA at the highest data rate. The difference between these two types of CSMA is hardly noticeable.

#### 7.1.2. Delay

**Figure 5:**
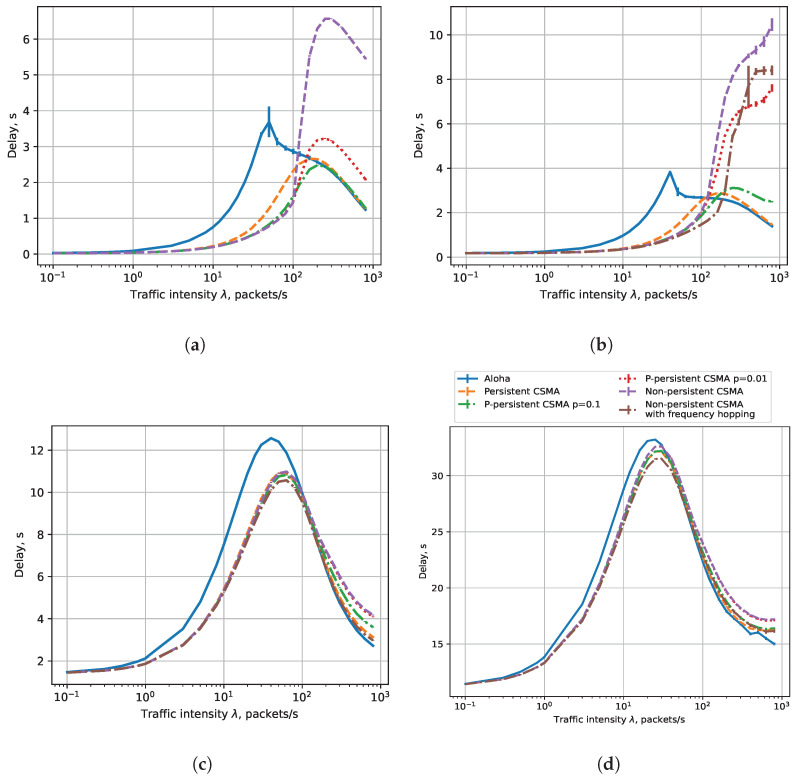
Delay, R = 0.4 km: (**a**) 25,600 bps, (**b**) 3200 bps, (**c**) 400 bps, (**d**) 50 bps.

Figure 5 shows the dependencies of the average delay on the load for six sensor operating modes.

Figure 5a,b show the average delay for the 25.6 kbps and 3.2 kbps rates, respectively. The results for the Aloha and CSMA modes are almost the same at low load. This happens because of the low probability of collision and the small time interval needed for carrier sensing in CSMA modes. At a load of about 2–100 packets/s, the average delay in the CSMA modes is much less than in the Aloha mode because a sensor in the Aloha mode wastes much time on retransmissions, while with CSMA, it has to wait for the channel to become idle but makes fewer retransmissions. At higher loads, the average delay in the Aloha mode is less than in the CSMA modes because packets are either transmitted successfully during the first attempt or are replaced by new ones, while in the CSMA modes, a sensor defers transmissions and avoids collisions. At a load below 100 packets/s, among different CSMA modes, non-persistent CSMA with and without frequency hopping obtain the lowest delay. With a higher load, the *p*-persistent CSMA with p=0.01, and non-persistent CSMA with and without frequency hopping have the highest average delay, while the persistent and *p*-persistent with p=0.1 ones have a lower average delay. Such a result contradicts [12] but is explained by the peculiarities of the buffering model because frames that wait too long in the buffer are preempted by the newer frames and are not taken into account in the statistics of the average delay (but are accounted for in the PLR).

Figure 5c,d show the average delay for the 400 bps and 50 bps rates, respectively. At low loads, the average delay is also almost the same for all the modes. At higher loads, the results differ from the rates of 25.6 and 3.2 kbps. With loads up to ≈40 packets/s for 50 bps and up to ≈100 packets/s for 400 bps, the average delay in the Aloha mode is higher than in CSMA modes. It happens because, in the Aloha mode, the sensors waste much time retransmitting packets after collisions, while in the CSMA mode, the sensors spend less time listening to the channel to avoid retransmissions. These results align with the classic studies of Aloha and CSMA, e.g., [12]. At higher loads, when PLR becomes almost the same in all modes, we see the opposite situation: in the CSMA modes, the sensor delays the transmission for a long time, while in the Aloha mode, the sensor transmits packets immediately after generation. Moreover, a notable difference between the CSMA modes can be explained by specific implementation features of each mode, such as the immediate start of the transmission in persistent CSMA or delaying the transmission in p-persistent CSMA and non-persistent CSMA. At high loads, in all modes, the average delay decreases because packets are frequently replaced by new ones, and successful packets are transmitted with few retries or without them at all. This result contradicts [12] but is explained by the peculiarities of the buffering model, i.e., that the packets are lost if new packets arrive before their transmission.

From the obtained results, we can conclude that the lowest delay is obtained with the data rate of 25,600 bps and non-persistent CSMA for loads up to 100 packets/s, and with p-persistent CSMA for loads greater than 100 packets/s. However, the loads above 100 packets/s are unfeasible for NB-Fi because the PLR becomes close to one at such loads.

#### 7.1.3. Throughput

**Figure 6:**
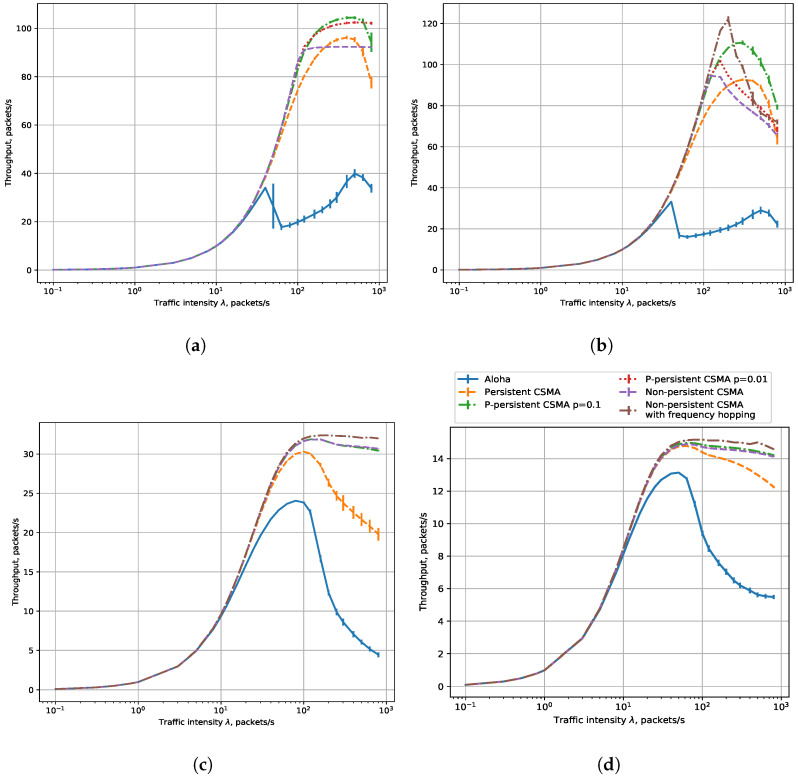
Throughput, R = 0.4 km, rates: (**a**) 25,600 bps, (**b**) 3200 bps, (**c**) 400 bps, (**d**) 50 bps.

Figure 6 shows the dependencies of throughput on load for six operating modes. We see that at low loads, the throughput plots for Aloha and CSMA modes almost coincide, which corresponds to the PLR results because almost all the packets are transmitted successfully through the channel and the throughput is equal to the load. At high loads, the difference in throughput is explained by the difference in PLR. A sensor in the Aloha mode loses most of its packets because of frequent collisions and consequently has the lowest throughput, while in the CSMA modes, listening to the channel makes transmissions more effective and results in a lower PLR and higher throughput. We model that sensors have a retry limit equal to 7, which means that the sensor discards its frame after making such a number of retries. However, increasing the retry limit does not change the PLR because the sensors very rarely reach it: with a much greater probability, they either transmit their frames or their frames are preempted by new frames. Similar to [12], among all the considered CSMA modes, the persistent CSMA achieves the lowest throughput, while the other kinds of CSMA achieve a comparable throughput. However, at very high loads (above 100 packets per second) and high data rates (3200 bps and 25,600 bps), we see that p-persistent CSMA with high *p* achieves the best performance, because at such loads, most packets are lost due to the buffer overflow, and delaying the frame transmission increases the PLR and decreases the throughput. Thus, we see that the buffering model provides results different from the classic results of CSMA.

As a strategy to obtain the highest throughput, we suggest using the data rate of 3200 bps and non-persistent CSMA with frequency hopping for loads up to 200 packets/s. For higher loads, we recommend using p-persistent CSMA with properly configured *p*.

#### 7.1.4. Energy Consumption per Successfully Transmitted Frame

**Figure 7:**
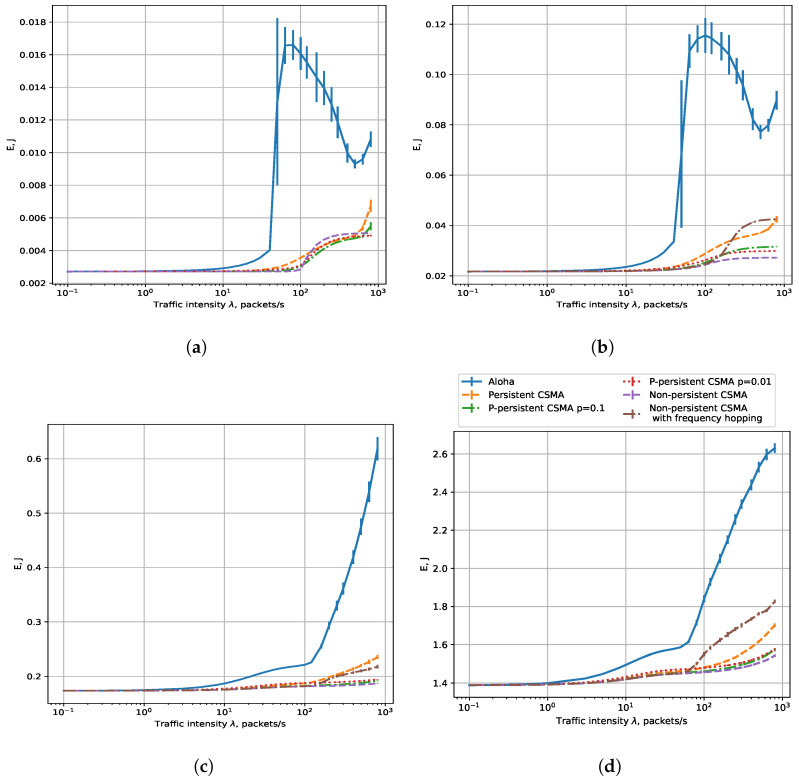
Energy consumption, R = 0.4 km, rates: (**a**) 25,600 bps, (**b**) 3200 bps, (**c**) 400 bps, (**d**) 50 bps.

Figure 7a shows the dependency of *E* on the load for a 25.6 kbps rate. We see that for all modes, *E* is almost the same at low loads because the sensors mostly transmit their frames at different times. At high loads, we see that Aloha and persistent CSMA become inefficient because of frequent collisions. In addition, we see that at high loads, the difference between different kinds of CSMA is not very high, but p-persistent CSMA with p=0.1 shows the best performance. This result is explained by the fact that at 25.6 kbps, all sensors use the same subchannel, which is mostly occupied, and the winning strategy is to wait for the channel to become idle and transmit after a random delay. At the same time, if the sensors wait too long, they lose their frames because of buffer overflows but waste energy on channel assessment, which is why non-persistent CSMA loses to p-persistent CSMA.

Figure 7b shows the dependency of *E* on the load for a 3.2 kbps rate. The energy consumption is also almost the same for all modes at low loads, but at high loads, collisions cause the sensors in the Aloha mode to retransmit their packets and thus waste much energy. In addition, the difference in energy efficiency between CSMA variants becomes greater due to different algorithms of listening to the channel and collision probability. Persistent CSMA is the least energy efficient because of the high collision probability (see PLR results in Figure 4a,b). After persistent CSMA, at high loads, we see that the energy consumption suddenly increases for non-persistent CSMA with frequency hopping, which is caused by frequent channel assessment and mostly occupied channels. The *p*-persistent CSMA with p= 0.1 exhibits the highest energy consumption, and the slightly lower energy consumption is seen when p= 0.01. Similarly to persistent CSMA, the sensor in the *p*-persistent CSMA mode wastes energy by sensing the channel and waiting until it becomes idle. When several sensors generate frames during someone else’s transmission, when the transmission ends, these sensors may attempt transmission, which will lead to a collision. The collision probability grows with *p*. Non-persistent CSMA is the most energy efficient because, in this mode, the sensor consumes the least amount of energy to listen to the channel while avoiding collisions.

Figure 7c,d show the dependency of *E* on the load for 50 and 400 bps rates, respectively. Again, the energy consumption for different modes is similar at low loads because (i) the sensors mostly transmit their frames at different times, and (ii) they rarely select overlapping subchannels for narrowband transmission. As a result, collisions are rare with Aloha, while with CSMA, the channel is usually idle and the sensor consumes little energy listening to the channel. At high loads, Aloha and persistent CSMA consume much more energy due to collisions. We also see that non-persistent CSMA consumes much energy at high loads because in this mode, transmissions are long and the channel is mostly occupied by transmissions of other sensors, so when a sensor switches from one subchannel to another one, it likely finds the channel busy and spends much energy on frequent channel assessment.

From the obtained results, we can conclude that the lowest energy consumption is obtained with a 25,600 bps data rate and non-persistent CSMA at loads up to 100 packets/s and p-persistent CSMA with properly configured *p* for higher loads.

### 7.2. Results for 3 km

Let us consider a wider network with an R= 3 km radius. In such a network, unlike an R=0.4 radius network, not all sensors can detect each other’s transmissions: sensors do not sense the transmissions of other sensors that are too far away, and thus can start their transmission even if some other sensors in the network transmit their data.

**Figure 8:**
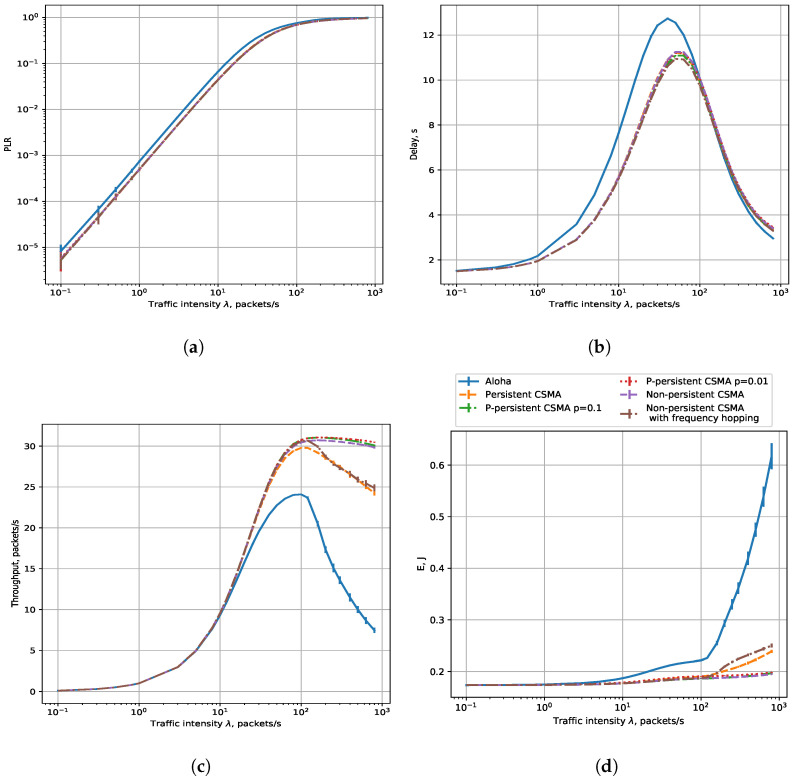
Performance evaluation results for R = 3 km and the data rate of 400 bps: (**a**) PLR, (**b**) delay, (**c**) throughput, (**d**) energy consumption.

The sensing range depends on the subchannel occupied by the signal, which is determined by the rate in NB-Fi (see Table 3), and for the rates of 50 and 400 bps, the range is comparable with 3 km, while for the rates of 3200 and 25,600 bps, it is significantly lower. For 50 and 400 bps, the sensors have a high transmission range. Moreover, at low rates, they are highly likely to use non-overlapping subchannels. Thus, for such data rates, the results are almost the same as those obtained for an R=0.4 network radius, see Figure 8, which is why we only show the results for the 400 bps rate. At the same time, for the rates of 3200 and 25,600 bps, the results are significantly different.

**Figure 9:**
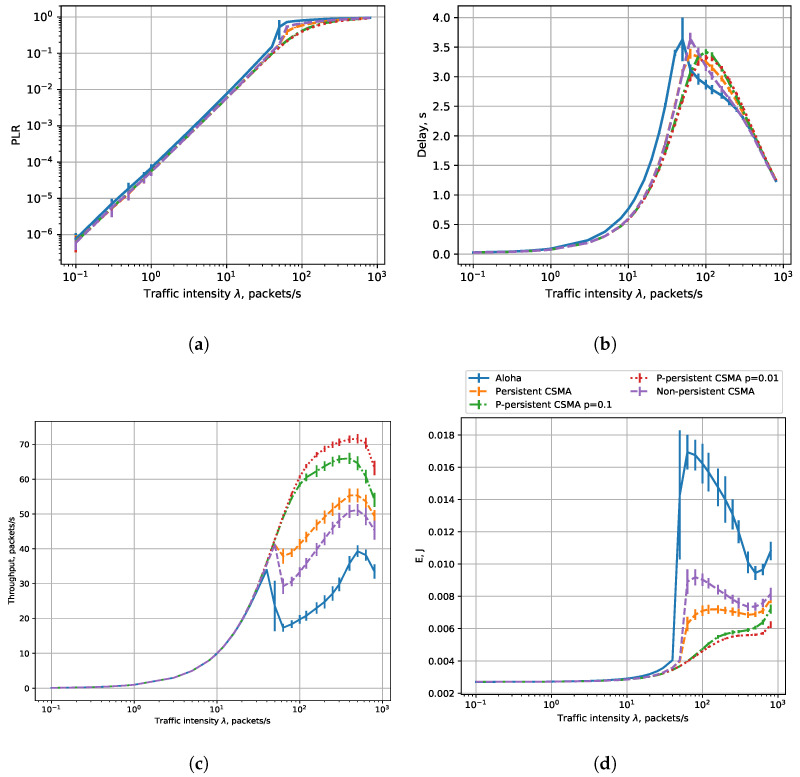
Performance evaluation results for R = 3 km and the data rate of 25,600 bps: (**a**) PLR, (**b**) delay, (**c**) throughput, (**d**) energy consumption.

Let us consider the results for the 25.6 kbps rate, where the differences between the 0.4 and 3 radius scenarios are the most notable. Figure 9 shows the dependence of PLR, delay, throughput, and energy consumption on load. First, the PLR in the CSMA mode does not differ so much from the Aloha mode. Among the CSMA modes, non-persistent CSMA appears to be the least efficient, while *p*-persistent CSMA shows the best results. It happens because, when the channel is idle, in the *p*-persistent CSMA mode, the sensor only transmits with the probability *p*. As a result, it transmits less frequently than when using non-persistent or persistent CSMA. We can also see the difference in average delay between the R=0.4 and R=3 scenarios. Results of the CSMA modes are more similar to the Aloha mode for R=3 than for R=0.4 because collisions happen with a higher probability. Throughput in all CSMA modes with a load of more than 40 packets/s becomes less than in the R=0.4 scenario, which happens due to more frequent collisions. A sensor’s energy consumption also increases due to collisions. We can also see that the energy consumption varies for different types of CSMA in intense traffic. Non-persistent CSMA is not as efficient as in the R=0.4 scenario anymore, while *p*-persistent has the lowest energy consumption, which corresponds to the throughput results.

To sum up, in a small network, to minimize PLR, the most efficient strategy is to use non-persistent CSMA and the highest data rate. The same strategy is efficient if we want to minimize the delay and energy consumption in a small network at a load below 100 frames per second. At higher rates, p-persistent CSMA with properly configured *p* is the best; however, at such loads, the PLR is close to 1. The maximal throughput is obtained with the non-persistent CSMA with frequency hopping and a 3200 bps rate. In a large network, the p-persistent CSMA is the most efficient for optimizing all the considered performance indicators.

## 8. Conclusions

This paper considers NB-Fi networks using LBT and non-LBT operation modes. We compared Aloha, persistent CSMA, non-persistent CSMA, non-persistent CSMA with frequency hopping, and *p*-persistent CSMA in terms of the PLR, average delay, throughput, and energy consumption of NB-Fi devices. We considered scenarios of a small network of radius R= 0.4 km, where all sensors can sense the transmissions of other sensors, and a wide network with an R= 3 km radius, where some sensors cannot sense transmissions of some other sensors.

Analysis of the simulation results shows that at low rates (50 and 400 bps), LBT achieves almost the same PLR as Aloha, which differs from the classic results of CSMA studies but is explained by the peculiarities of NB-Fi modulation. At rates of 3200 and 25,600 bps in a small network, LBT has a much greater effect; in a large network, the effect is smaller. At high loads, the persistent CSMA always has the highest PLR results among other types of CSMA and should not be used. Note that in a network of a small radius, it is better to use the highest data rate with non-persistent CSMA, while in a network of a large radius, it is better to use *p*-persistent with small *p*.

At low data rates, the average delay in LBT modes is a little different from the average delay in the Aloha mode. At a high rate in a small network with a high load, the average delay when using the carrier sensing mode noticeably exceeds the average delay when operating in the Aloha mode. In a large network, the difference in the average delay at high loads becomes smaller. These results contradict the classic studies on CSMA but are explained by the buffering model, which more accurately describes the IoT devices. For feasible loads, the best delay is obtained with the highest data rate and non-persistent CSMA in a small network and p-persistent CSMA in a large network.

As predicted by classic papers, CSMA at high loads significantly increases throughput. At the same time, *p*-persistent and non-persistent CSMA modes with frequency hopping have the highest throughput at high loads.

As can be expected, at a load below one packets/s, the use of CSMA does not reduce the energy consumption compared to Aloha. At high loads (more than 40 packets/s), CSMA can lower the energy consumption more than twice in comparison with Aloha. Among the considered types of CSMA, persistent CSMA has the highest energy consumption at a high load. At the same time, at high loads, non-persistent CSMA shows the lowest energy consumption in networks of small radius, while *p*-persistent CSMA with small *p* is the most effective in networks of large radius.

The practical result of this research is the recommendation to use the non-LBT mode in NB-Fi networks with low loads or in NB-FI networks where devices operate only at low data rates (50 bps or 400 bps), while at higher loads and higher data rates, the best practical solution could be to use non-persistent CSMA in small networks, where all devices can sense each other’s transmissions, and to use p-persistent CSMA with an optimized *p* parameter in large networks.

We see several directions for future work. The first one is studying the performance of NB-Fi in multi-BS scenarios. The second direction is studying approaches to satisfy the quality of service (QoS) requirements for different kinds of traffic that are generated in the same NB-Fi networks. The third direction is to develop rate and power control algorithms that can decrease energy consumption, PLR, and delay in such networks. An important subcase of this problem is when the devices are mobile. Finally, the coexistence of NB-Fi networks with networks of other technologies operating in ISM bands should also be studied. 

## Figures and Tables

**Table 1:** Summary of the literature review.

Paper(s)	Main Contribution	Difference with This Research
[19]	Review and comparison of nominal parameters of such LPWAN technologies as LoRa, Sigfox, NB-Fi, NWave, and RPMA.	Does not study LBT in NB-Fi.
[20]	Description of a structure of an IoT device that uses NB-Fi or other LPWAN technology.	Does not study LBT in NB-Fi.
[4,21]	A thorough study of NB-Fi standard and performance evaluation in a scenario with numerous sensors.	Do not study LBT in NB-Fi.
[15]	Evaluation of energy consumption of devices in NB-Fi networks where devices use LBT	The power consumption of devices does not correspond to the device specification [34]. Does not consider PLR, delay, and throughput.
[12,23,24,25,26,27]	Performance evaluation of LBT and non-LBT modes for networks of different technologies.	Do not compare the devices’ energy consumption and do not consider the peculiarities of NB-Fi.
[28,29]	Compare the device energy consumption in the Aloha and “non-persistent CSMA” modes for LoRaWAN networks.	Do not study LBT in NB-Fi. Do not study p-persistent CSMA.
[30]	Propose to use *p*-persistent CSMA in LoRaWAN.	Do not study LBT in NB-Fi. Consider scenarios with only a small number of devices.
[31,32,33]	Develop new LBT mechanisms for LoRa that improve the channel efficiency.	Do not study LBT in NB-Fi. Do not compare these mechanisms with non-LBT.
[22]	Comparison of NB-Fi, LoRaWAN, and Sigfox in a wide range of scenarios.	Does not study LBT in NB-Fi.

**Table 2:** Time intervals.

Data Rate *r*, bps	$T_{\mathrm{Frame}}$, ms	$T_{\mathrm{delay}}$, ms	$T_{\mathrm{listen}}$, ms	$T_{\mathrm{rnd}}$, ms
50	5760	140	60,000	5000
400	720	20	30,000	1000
3200	90	5	6000	100
25,600	11.25	3.75	6000	100

**Table 3:** Network and device characteristics.

Data Rate, bps	σ, µs	*S*, dBm	$R_{\mathrm{sensor}}$, m	$R_{\mathrm{base}}$, m
50	20,000	−150	4270	12,150
400	2500	−141	2460	7650
3200	312.5	−132	1420	4810
25,600	39.06	−123	820	3030

**Table 4:** Sensor energy consumption [34].

Description	Notation	Value
Current in receiving state	$I_{rx}$	17 mA
Current in transmitting state	$I_{tx}$	50 mA
Current consumed by the power source	$I_{s}$	3 mA
Voltage	*V*	3.3 V
Power consumed for transmission	$P_{tx}$	175 mW
Power consumed for sensing and reception	$P_{rx}$	66 mW

## Data Availability

No new data were created or analyzed in this study. Data sharing is not applicable to this article.

## Abbreviations

The following abbreviations are used in this manuscript:

BS	Base station
CSMA	Carrier Sense Multiple Access
DBPSK	Differential binary phase-shift keying
DL	Downlink
DRX	Discontinuous reception
IoT	Internet of Things
ISM	Industrial, Scientific, and Medical
LBT	Listen Before Talk
LPWAN	Low-power wide-area network
NB-Fi	Narrow Band Fidelity
PLR	Packet loss rate
PER	Packet error rate
QoS	Quality of service
SINR	Signal to interference and noise ratio
UL	Uplink
